# The Reasons for Self-Medication from the Perspective of Iranian Nursing Students: A Qualitative Study

**DOI:** 10.1155/2022/2960768

**Published:** 2022-04-06

**Authors:** Maryam Janatolmakan, Alireza Abdi, Bahareh Andayeshgar, Ali Soroush, Alireza Khatony

**Affiliations:** ^1^Social Development and Health Promotion Research Center, Health Institute, Kermanshah University of Medical Sciences, Kermanshah, Iran; ^2^School of Nursing and Midwifery, Kermanshah University of Medical Sciences, Kermanshah, Iran; ^3^School of Health, Kermanshah University of Medical Sciences, Kermanshah, Iran; ^4^Cardiovascular Research Center, Health Institute, Kermanshah, Iran; ^5^Infectious Diseases Research Center, Kermanshah University of Medical Sciences, Kermanshah, Iran

## Abstract

**Background:**

The prevalence of self-medication has increased dramatically worldwide. This study was conducted to determine the reasons for self-medication from the perspective of Iranian nursing students.

**Methods:**

This qualitative study was conducted using the content analysis method. Fifteen nursing students were selected by the purposeful sampling method. Data were collected by in-depth semistructured interviews. Qualitative content analysis method was used for data analysis. The MAXQDA software was used for data management.

**Results:**

Data saturation was achieved with fifteen interviews with nine women and six men, with a mean age of 26.5 ± 4.8 years. The reasons for self-medication were explained in five categories and fifteen subcategories. Some of the reasons for self-medication were having medication information, having previous experience, easy access to medicine, lack of enough time, access to medical staff, cost of a doctor's visit, inadequate respect for patient privacy, pharmaceutical advertising in the media, and information explosion. *Discussion*. Several factors are involved in self-medication. Given the dangers of self-medication, health policymakers must adopt strict policies for pharmacies that sell drugs without a prescription. Furthermore, it is helpful to run training courses on self-medication risks for students.

## 1. Introduction

Self-medication refers to the use of one or more medications without a physician's prescription [1]. It is one of the health problems in many countries, including Iran [2]. It has been viewed as one of the main social issues on economic and health systems with variety in prevalence based on contextual determinates such as education level, knowledge, income, and gender [1, 2]. The prevalence of self-medication has increased dramatically worldwide. Evidence suggests that about 80% of all medications in developing countries can be bought without prescriptions [3]. Self-medication, due to its lower cost, is a substitute for people who cannot afford proper medical care [4]. Today, the arbitrary use of drugs is increasing among the young people, especially students, and the probability of self-medication is greater among nursing students because of their easy access to information sources and their relative familiarity with different drugs [5]. Evidence suggests that the prevalence of self-medication is relatively high among medical science students in different countries. In this regard, the prevalence of arbitrary drug use has been reported to be 78.6% in India, 76% in Brazil, 78.5% in Jordan, 97.5% in Kuwait, and 70.1% in Iran [1, 6–9]. Major problems associated with self-medication include adverse drug reactions, drug resistance, waste of resources, and serious health risks such as death [1, 10]. There is also the possibility of incorrect treatment due to misdiagnosis of the disease [4]. There are numerous reasons for self-medication. In this regard, the results of a study in Ethiopia showed that previous experience, access to pharmacies, family pressure, and availability of medications at home were among the reasons for self-medication in medical science students [10]. The mildness of the disease and previous knowledge about medications are among other reasons for self-medication [2, 3, 11].

The results of a study in Bangladesh showed that education and socioeconomic status had a great impact on self-medication [12]. Most studies conducted on the reasons for self-medication from the perspective of students are quantitative. As self-medication is largely related to the subjective factors, qualitative methods such as surveys can identify the individuals' experiences and rationales that otherwise cannot be determined by quantitative methods [13]. Therefore, due to the lack of knowledge on the reasons for self-medication in nursing students, this study was conducted to explain the reasons for self-medication from the perspective of nursing students.

## 2. Methods

### 2.1. Design

The conventional qualitative content analysis approach, based on the Graneheim and Lundman's method, was used to undertake this qualitative research [14].

### 2.2. Participants and Sampling Method

The sample consisted of 15 undergraduate and graduate nursing students who were recruited by purposive sampling. To increase the richness of the data, and in line with the rigor principles, the participants were selected from both sexes, different age groups, and undergraduate and graduate students. The inclusion criteria consisted of having a history of self-medication, consent to participate in the study, and employment in the field of nursing. It should be noted that the samples of this study included those who had a history of self-medication based on the results of a cross-sectional study on the prevalence of self-medication [2] and expressed their consent to participate in future studies by providing e-mail and phone number. Sampling was carried out until the data were saturated.

### 2.3. Setting

This study was performed at Imam Reza Hospital Complex in Kermanshah, Western Iran.

### 2.4. Data Collection

The data were collected by semistructured interviews. The interviews were conducted in the researcher's room, located in Imam Reza Hospital Complex. The researcher began the interview with guiding questions like “What are your reasons for self-medication?” and “What factors prevent you from visiting a doctor?” The researcher then used phrases such as why, how, and please explain more to clarify the issue. All interviews were recorded by using an audio recorder. Each interview lasted 30–50 minutes. Interviews were continued until data saturation was accomplished. Data saturation occurs when adding an interview does not produce any new information [15]. In the fifteenth interview, the data were saturated. Interviews were conducted by the first author, a woman with a master's degree in nursing and 18 years of clinical and educational experiences.

### 2.5. Data Analysis

Data analysis and collection were performed simultaneously. After each interview, the participants' statements were transcribed and then fed into the MAXQDA-2013 software [16]. This software was used for initial data coding. The texts of the interviews were then read several times. Meaning units were then extracted, condensed, and labeled. At this stage, the subcategories were emerged. In the next step, the categories were determined based on differences and similarities [17]. The classification of primary codes and their relationship with new data and the formation of subcategories and categories were carried out by the research team on a regular basis. To confirm the coding process, the codes, categories, and subcategories were provided to four nursing students, who approved them.

### 2.6. Trustworthiness

Denzin and Lincoln have proposed four criteria of credibility, transferability, dependability, and confirmability for assessing interpretive research [18], which were used in this study. To increase the credibility of the data, the researcher tried to convey the feeling of comfort and security to the participants, so that they could easily talk about and explain the reasons for self-medication. Moreover, the texts of interviews were shared with the participants for more certainty. To enhance the transferability of the results, the phenomenon of self-medication was thoroughly investigated, and the participants' characteristics such as age and sex were described. The results of this study were also given to four students, including two undergraduate and two graduate students to compare with their experiences in order to confirm the codes, subcategories, and categories. To increase the dependability of the data, all stages of the study were described step by step to be easily verified during external auditing. To increase the confirmability of the data, some of the interviews and transcriptions along with the coding were provided to two research colleagues who were expert in qualitative research and were not part of the research team in order to confirm the accuracy of the coding process.

### 2.7. Ethical Considerations

The Ethics Committee of Kermanshah University of Medical Sciences approved the study. The objectives of the study were explained to all participants, and informed written consent was obtained from all of them. Participants were reassured about the anonymity and confidentiality of their personal information. To protect anonymity, participants' identical information was coded in written documents as well as when sent to other researchers and external audit. The audio files were also saved by the corresponding author in a file with a username and password. In addition, the personal information of the participants is not mentioned in the paper, and this data will be protected forever by the corresponding author.

## 3. Results

The mean age of the participants was 26.5 ± 4.8 years. Seven of them were undergraduate students and eight of them were postgraduate students. Furthermore, nine of them were female and six were male. After analyzing the data, five main categories and fifteen subcategories were developed. The main categories included individual factors, economic factors, physician-related factors, culture, and media impact (Table 1).

## 4. Individual Factors

In the present study, the individual factors related to self-medication were expressed in six subcategories, including “having medication information, having previous experience, easy access to medicine, not having enough time, access to medical staff, and not taking the disease seriously.”

### 4.1. Having Medication Information

All participants considered their field of study one of the reasons for self-medication and believed that they were familiar with illnesses and medications through their nursing education. They also believed working with physicians and clinicians increased their awareness of the treatment of many illnesses. One of the participants in this regard stated *“My field of study affects my self-medication because I study extensively. Also, as we work with physicians, we use their experiences”* (participant no. 4).

Another participant said *“Nursing students are familiar with a variety of diseases and medications, and this is one of the reasons for self-medication. Passing four credits of pharmacology in the first semester is effective in self-medication”* (participant no. 14).

### 4.2. Having Previous Experience

Having a positive or negative previous experience of self-medication is an important factor in performing self-medication. This experience can be related to the person or his/her friends and relatives.

*“When I go to a doctor for a particular problem and receive certain medications, I use the same medications next time I develop the same problem and have the same symptoms”* (participant no. 15).

*“…. if my friends or relative who have the same symptoms recommend me to use certain medications, I will use them because I know that they have used them before and have become well”* (participant no. 2).

### 4.3. Easy Access to Medicine

The sale of medication by pharmacies without a doctor's prescription is one of the main reasons for self-medication. Unfortunately, some pharmacies provide the requested medication to clients without informing them about the risks of self-medication.

A participant in this regard stated “*One of the reasons for self-medication is easy access to medicine in the pharmacy”* (participant no. 8).

Another interviewee commented *“The availability of medications affects self-medication, and currently the sale of over-the-counter medication is excessive. When medications are available, people may easily buy and take them on others' recommendation”* (participant no. 5).

### 4.4. Not Having Enough Time

Nursing students have an intensive curriculum from morning to evening and do not have enough time to visit a doctor or go to a medical center, so they may opt for self-medication.

One of the participants said:

*“I attend classes from morning to evening, and if I get sick, I do not have a chance to see a doctor*” (participant no. 1).

*“Many doctors are in the office until 8 pm, and it is closed when I get to the office from school”* (participant no. 12).

### 4.5. Access to Medical Staff

One of the reasons that can play a role in self-medication is the presence of a nurse or doctor among the family members, friends, and relatives. However, these doctors and nurses may be consulted due to their knowledge of various diseases.

*“My friend is a specialist. Whenever I have a question about the treatment of a certain disease, I ask him”* (participant no. 5).

### 4.6. Not Taking the Disease Seriously

A person's attitude toward health is effective in his/her treatment process. Unfortunately, someone who does not value his/her health does not take his/her illness seriously and tries to self-medicate.

*“Some students do not take their illness seriously and therefore try to self-medicate”* (participant no.3).

*“Some do not take their illness seriously at all and try to treat it arbitrarily”* (participant no. 5).

## 5. Economic Factors

There is a close relationship between economic status and the desire for self-medication. Inadequate economic situation can be the basis of self-medication, which was mentioned in the statements of all participants. This category included the subcategories cost of a doctor's visit, cost of paraclinical services, and cost of travel.

### 5.1. Cost of a Doctor's Visit

In Iran, some doctors do not have an insurance contract, and the patient has to pay for the visit out of pocket. However, for those doctors who have a contract with insurance companies, their visit costs a significant amount. For this reason, they preferred to treat themselves rather than to see a doctor.

*“The cost of a doctor's visit is very high and my income is limited, this prevents me from visiting a doctor, and I have to do self-medication”* (participant no. 4).

*“Many doctors do not have insurance contracts, and we have to pay all the cost for the visit out of our own pocket, which is a significant amount”* (participant no. 2).

### 5.2. Cost of Paraclinical Services

Sometimes laboratory tests and radiographic examinations are needed to make a medical diagnosis, which are expensive. Some participants stated that in addition to the cost of visit, they also have to pay for paraclinical services; therefore, they do not go to see a doctor and do self-medication instead. One of the participants stated:

*“Many doctors do laboratory and radiology studies such as ultrasonography to diagnose the disease, which costs me a lot as a student”* (participant no. 1).

*“It's not just the visit cost, laboratory tests, radiography, and ultrasound are also too costly”* (participant no. 11).

### 5.3. Travel Cost

The cost of commuting to doctors' offices or medical centers is high, which can lead to patients' use of self-medication. One participant stated: *“Someone who goes to the doctor is forced to travel across the city and spend lots of money on transport”* (participant no. 13).

Another participant said: *“I have to spend time and pay for travel expenses to visit a doctor, I do self-medication for simple illnesses instead*” (participant no. 9).

## 6. Physician-Related Factors

Self-medication was found to be partly associated with doctors. It included subcategories crowded doctors' offices, inadequate respect for patient privacy, and doctors' low-quality visit.

### 6.1. Crowded Doctors' Offices

The crowded doctors' offices and medical centers were another factor that was mentioned by the participants.

*“It takes several months to visit some specialists, and the patient has no choice but to self-medicate”* (participant no. 3).

*“Many doctors' offices are so crowded that I have to wait for hours, so I have no choice but to self-medicate”* (participant no. 7).

### 6.2. Inadequate Respect for Patient Privacy

Patient privacy should be a physician's priority, but some participants believed that this was not the case in some physicians' offices.

*“I used to have too much acne, so I went to a skin clinic. When I arrived, students encircled me and I felt very bad. I felt like a lab mouse. It was very unpleasant for me; such things make you do self-medication”* (participant no. 9).

*“In some doctors' offices, a secretary sends two or three patients to the doctor's room at the same time! How am I supposed to talk about my sexual organs in front of others? These make the patient self-medicate rather than go to visit a doctor”* (participant no. 10).

### 6.3. Doctor's Low-Quality Visit

Clinical examination is an important part of the diagnosis process. Some participants believed that some physicians do not examine the patient at all and only rely on a variety of expensive tests and high-resolution imaging for diagnosis. This factor leads to patients' reluctance to visit a doctor, so they perform self-medication.

*“Some physicians only take into consideration the patient's complaints and do not examine him/her”* (participant no. 8).

Another participant stated *“Some physicians prescribe a comprehensive set of tests and imaging in the first visit and do not perform any physical examination, which is an important part of medical practice”* (participant no. 6).

## 7. Cultural Factors

Another factor that contributed to the participants' self-medication was culture. This category was mentioned by the participants and included a subcategory entitled acceptance of self-medication in society.

### 7.1. Acceptance of Self-Medication in Society

Self-medication is closely related to the culture of society. In the Iranian society, self-medication is common and accepted, and a significant proportion of Iranians think that medicine is needed for any particular disease. Others think that the other persons' disease is similar to theirs; therefore, they arbitrarily take the same drugs.

*“In society, if someone gets sick, he/she first seeks treatment from friends and acquaintances, and people not only do not have a bad attitude toward this type of behavior, but also recommend various treatments to him/her”* (participant no. 3).

*“Unfortunately, self-medication is a socially accepted behavior, and people who recommend medication to others are considered literate”* (participant no. 7).

## 8. Media Impact

All participants repeatedly mentioned the role of mass media in promoting the phenomenon of self-medication. This category included two subcategories entitled pharmaceutical advertising and information explosion.

### 8.1. Pharmaceutical Advertising

Mass media have a profound effect on the members of society, but they are like a double-edged sword that can either make the public aware of the dangers of self-medication or take steps to spread the ominous phenomenon of self-medication by distributing the advertisements of various pharmaceutical companies.

“Satellite networks, newspapers, and magazines play an important role in encouraging people to use a variety of chemical and herbal remedies for weight loss, height growth, and so on” (participant no. 2).

### 8.2. Information Explosion

Today, information explosion is occurring in all fields, including medical knowledge. A simple search on Google can yield a large amount of information about various diseases and their treatments. All participants reported they had easy access to medical information through the media, especially the Internet.

*“When I get sick, I go online and search my illness, and according to the symptoms, I take medications”* (participant no. 8).

## 9. Discussion

The purpose of this study was to explain the reasons for self-medication from the perspective of Iranian nursing students. The results showed that one of the reasons for self-medication was related to individual factors, including having medication information, having previous experience, easy access to medicine, not having enough time, access to medical staff, and not taking the disease seriously. Previous studies have reported reasons such as previous self-medication experience [2, 9, 19–21], availability of drugs [2, 10, 22–24], having sufficient medical knowledge [2, 9, 25–27], lack of time [2, 24, 28], access to medical staff [2], and non-seriousness of the disease [2, 24] for self-medication. However, it should be noted that there is a possibility of misdiagnosis by students that can exacerbate the risks of self-medication [4].

Another reason for self-medication from the perspective of participants was economic factors. In this regard, the participants mentioned reasons such as cost of a doctor's visit, cost of laboratory tests, and transportation cost, which are in line with previous studies [2, 20, 29–31]. Students are financially dependent on their family and may not be able to pay for their treatments, which is a great incentive for self-medication.

Another reason for self-medication was related to the physicians. In this regard, the participants noted reasons such as crowded offices, lack of patient privacy, and poor quality of visits. Previous studies have also reported reasons such as distrust in the physician's diagnosis [25, 32, 33], crowded physicians' offices [4, 9, 33], and lack of attention to patient privacy [2, 34, 35].

Cultural reasons for self-medication were also reported by the participants, which included the subcategory of acceptance of self-medication in society. The belief in the indiscriminate use of various medicines as well as the safety of self-medication is rooted in the culture of the society. In this regard, evidence indicates that the cultural context is one of the reasons for self-medication [12, 36]. The results of a systematic review showed that the main reasons for self-medication with antibiotics in the Middle East were sociocultural factors and economic reasons [21].

### 9.1. Study Limitation

Due to the impact of various factors, including cultural, social, and economic, on self-medication, the generalizability of the results is limited.

## 10. Conclusion

The results showed that social media such as the Internet and satellite play a role in self-medication, and following drug advertisements and online information can be dangerous. Mass media play an important role in promoting public health by institutionalizing the culture of visiting a physician instead of performing self-medication. Given the dangers of self-medication, the health policymakers must adopt strict policies for pharmacies that sell over-the-counter drugs. Developing training programs to change nursing students' attitudes toward self-medication can also be helpful. Similar studies are recommended to be conducted on students of different disciplines. However, self-medication is a common issue among nursing students and should be tailored to suit any context. In this regard, researchers can examine the effects of intervention measures on the students' self-medication rate by considering the reasons for self-medication.

## Figures and Tables

**Table 1:** Categories and subcategories related to the nursing students' perspectives about the reasons for self-medication.

Categories	Subcategories
Individual factors	Having medication information
	Having previous experience
	Easy access to medicine
	Not having enough time
	Access to medical staff
	Not taking the disease seriously

Economic factors	Cost of a doctor's visit
	Cost of paraclinical services
	Travel cost

Physician-related factors	Crowded doctors' offices
	Inadequate respect for patient privacy
	Doctors' low-quality visit

Cultural factors	Acceptance of self-medication in society

Media impact	Pharmaceutical advertising
	Information explosion

## Data Availability

The identified datasets analyzed during the current study are available from the corresponding author on reasonable request.
